# Metacognitive Awareness and the Hot Hand: When Winning, No Amount of Awareness Will Have Strong Believers Avoid the Heuristic

**DOI:** 10.3390/jintelligence11070149

**Published:** 2023-07-22

**Authors:** Yeonho Choi, Lisa K. Son

**Affiliations:** Department of Psychology, Barnard College, New York, NY 10027, USA; lson@barnard.edu

**Keywords:** hot hand fallacy, metacognition, awareness, heuristics, decision-making

## Abstract

In some instances, such as in sports, individuals will cheer on the player with the “hot hand”. But is the hot hand phenomenon a fallacy? The current research investigated (1) whether the hot hand fallacy (HHF) was related to risky decisions during a gambling scenario, and (2) whether metacognitive awareness might be related to optimal decisions. After measuring for baseline tendencies of using the hot hand heuristic, participants were presented with a series of prior card gambling results that included either winning streaks or losing streaks and asked to choose one of two cards: a good card or a bad card. In addition, we examined whether high metacognitive awareness—as measured by the ability to discriminate between correct and incorrect responses—would be negatively related to the risky decisions induced by the hot hand heuristic. The results showed that our predictions were partially supported. For winning streaks, individuals who had a weak tendency for using the heuristic exhibited fewer risky decisions with higher metacognitive awareness. However, those with a strong baseline tendency for using the hot hand showed no sign of decrease with metacognitive awareness. On the whole, the complex data suggest that further research on the HHF would be helpful for implementing novel ways of avoiding the fallacy, if needed.

## 1. Introduction

*Consider the following series of coin tosses: H-T-H-H-H. If the same coin were tossed once again, what would you predict, H or T? Would the past pattern of prior results affect your prediction?*

Despite being tossed repeatedly, the results of each coin toss are independent from one another. However, research has shown that individuals will often form a pattern between the toss results to inform their prediction (Roney and Trick 2009). Two types of biases in particular have been found consistently and in different contexts: (1) the gambler’s fallacy and (2) the hot hand fallacy. The gambler’s fallacy (GF) refers to the expectation that any event which was predominant in the past (H in the example above), would be reversed (prediction would be T); the hot hand fallacy (HHF) refers to the expectation that any “hot” event, presumably having acquired some kind of momentum, is likely to re-appear (an H would be predicted as there had been a streak of Hs just prior). Any individual with either of the above biases would be at risk of making decisions that are non-optimal. The current research investigated the severity of the HHF when considering risky events, as well as the potential for metacognitive awareness in helping gamblers elude the fallacy.

## 2. The Hot Hand Fallacy

Knowing who has the “hot hand” is crucial, especially when participating on a team sport. Teammates have to make momentary decisions during a game, and the optimal pass would be to get the ball to the player who is “on fire”. Even fans in the stands might judge that a particular ball player had the hot hand that night, and believing that they could advise the players, could scream from the stands, “Throw it to Curry, for God’s sake!” Players, fans, and coaches alike make ongoing assessments of whether a particular player is hot, and these assessments will guide their decisions. But is the phenomenon real? Can someone have the hot hand, and do people believe that one can be on fire? 

Gilovich et al. (1985) first investigated the hot hand in the context of basketball through four experiments. Although they found that basketball fans made predictions that aligned with the hot hand (Experiment 1), they also found, statistically, that the hot hand was not existent. That is, they showed that a prior shot did not systematically affect the player’s chances of making the next shot. This fallacy was found when looking at NBA team data (Experiment 2), when analyzing the Boston Celtic’s free-throw data (Experiment 3), and when examining Cornell’s basketball game data (Experiment 4). In other words, making a successful shot was not more likely to be followed by a basket than a miss. Thus, the researchers dubbed the hot hand belief a fallacy, resulting in what is now known as the HHF. This fallacy was defined by the process of mistaking chance events as being influenced by prior outcomes. Put simply, the HHF stated that individuals perceived streaks where there were none (Burns and Corpus 2004).

What is the mechanism that leads to the HHF? Gilovich et al. (1985) suggested that the HHF occurs because people may perceive a random event as having been planned, such as when a “random” streak occurs. When a streak occurs, it can happen under random circumstances, but people may put a lot of weight on the streak, expecting that the positive correlation will continue. This type of explanation could also explain the reason for the occurrences of the representative heuristics or the law of small numbers (Ayton and Fischer 2004; Burns and Corpus 2004; Tversky and Gilovich 1989). Simply, streaks allow people to perceive random events as non-random, especially when the streaks come from a small sample, distancing the results from what is seen in the larger population (Ayton and Fischer 2004; Burns and Corpus 2004).

While the HHF describes a bias, in reality, we can acknowledge that there may be reasons for why believing in the hot hand may be positive. The HHF may be an adaptive heuristic. In the least, using heuristics works as a way in which to save cognitive resources when faced with a complex problem (Gilovich et al. 2002). Using full and deliberate thinking, on the other hand, may be inefficient. Further, especially in the context of sports, supporting your players by believing in them may, in fact, be a way to “put them on fire,” even if they might not have actually been on fire. It may be another way in which to express the common remark, “You got this!” Particularly for fans, then, the HHF may appear because of confirmation bias. That is, when fans expect that their player will be hot, they will be looking for the shots where they are hot. And whether or not the hot hand is a fallacy may not be important. In the end, both the players and fans feel good, and the existing fans become bigger fans. It is not surprising, then, that people will not hesitate from using the hot hand heuristic in certain situations.

Indeed, the notion that the hot hand is a fallacy is not crystal clear when it comes to sports. There have been a few more recent studies that have claimed that there do appear to be “hot streaks” at times (Miller and Sanjurjo 2018; Yaari and Eisenmann 2011). For example, Miller and Sanjurjo (2018) argued that Gilovich et al.’s study had bias in the data selection procedure because they looked only at the data that followed a series of hits or misses. This showed that data such as those could underestimate the expected probability of successes as compared to the true probability of successes. Using a novel procedure, they were able to reveal, after correcting the bias, that the hot hand phenomenon would be apparent in Gilovich et al.’s study. In other words, players who made successive shots before would be more likely to succeed in making the subsequent shot. Thus, it may be that, in the context of sports, and because of the various perspectives of analyses, an individual player or players could very well possess the hot hand. 

The case would be different for scenarios that do not include fans and where motivational utterances would be irrelevant—such as when gambling. And one might predict that perhaps the hot hand heuristic would not be used in such situations. However, that is not the case. The hot hand heuristic has been commonly found even in gambling contexts, despite potentially leading to biases that can lead to detrimental results (Suetens et al. 2016; Sundali and Croson 2006). Sundali and Croson (2006) analyzed field data from a casino, using gambling data from the roulette table. They found that after the ball had landed on a specific number, some gamblers bet more on that same number while other gamblers bet less on that number—even though the expected probability of the roulette wheel outcomes is fair—describing both the HHF and the GF, respectively. Suetens et al. (2016) also found a similar result. They showed that people succumbed to both the GF, betting less on a number that appeared in the previous week’s lotto, and the HHF, betting more on a “hot number” that had been drawn several times before. In these circumstances, there can be no hot hand, as the drawings are unrelated and could lead the “better” to make rash decisions linked to grim fiscal consequences. 

Camerer (1989) argued that the HHF is especially critical in that it is likely to be related to the way in which people allocate their resources. In fact, several negative consequences stemming from the HHF were found in previous studies. Johnson et al. (2005) found that the HHF could be related to irrational decision-making in the stock market: people tended to buy stocks when they were rising and, on the flipside, tended to sell when stocks were falling. This strategy not only is incompatible with the optimal stock market strategy, “Buy Low-Sell High,” but will also undoubtedly be related to potential loss and regret. If people misunderstand the relationship between stock-price trends and future prices, and react based on the HHF, they might not then gain a greater profit. For example, they might display the “overreaction” phenomenon, mistaking stock with the worst return as continuing in the same fashion (Offerman and Sonnemans 2004) when, in reality, a falling stock, not a rising stock, could give the best returns in the future. 

In addition to the reality that people use the hot hand heuristic in situations that underscore its fallacious nature, the HHF has also been shown to be potentially related to psychological and pathological problems. As indirect evidence, Leonard and Williams (2016) investigated the relationship between the fallacies, pathological gambling, and impulsivity and found that one’s fallacy score (which was higher when one avoided the fallacies) was negatively correlated with pathological gambling, gambling frequency, impulsivity, and so on. Meanwhile, Dohmen et al. (2009) argued that the HHF could affect economic status, including unemployment. They found that people who tended to succumb to the HHF were at higher risk of long-term unemployment. Presumably, people who based their decisions on the HHF overrated any experience of failing to find employment and, consequently, believed that they would fail to get a job (Dohmen et al. 2009). Taken together, the HHF could have serious consequences, and our goal was to find ways in which to help individuals escape the HHF. In the current paper, we focused on the idea that having more awareness would be helpful in avoiding such biases. Below, we discuss related background from the literature on metacognition. 

Metacognition, or the awareness of one’s own cognitive process, comprises two distinct components, monitoring and control, which together act as one big feedback loop that allows for constant updating and adjusting during decision-making (Nelson and Narens 1990, 1994). The updating process allows us to have better performance in various fields including decision-making. For instance, Batha and Carroll (2007) showed that mere metacognitive instruction allowed participants to make more optimal decisions. Specifically, researchers gave not only a task to measure decision making abilities that required to develop orders of appropriate plans to complete specific business scenarios but also metacognitive instruction (e.g., “Ask yourself what information you need to make a decision,” or “Revise your computation to check for errors or missed information,” etc.) to the experimental group after completing a pre-test to measure decision-making ability. They found that, as compared to the control group, the metacognitive instruction group was better at designing the orders of business plans, indicating a higher level of decision-making skill. This finding does not point to the specific mechanism as to how people make more optimal decisions but suggests simply that some level of awareness seems necessary for avoiding certain fallacies. We were interested to see whether metacognitive awareness would be associated with the hot hand heuristic in particular, which, if true, would be in line with data by Whitson and Galinsky (2008), which showed that lack of control made people perceive illusory patterns in situations with no consistent pattern or algorithm. Their results revealed that perceiving one’s own thinking and controlling it accordingly are crucial when avoiding specific heuristics.

Several studies have found that increasing metacognitive awareness, through simple instruction or training, can lead to more adaptive behavior, either through increased monitoring or more optimal regulation. For example, Gawęda et al. (2015) found that metacognitive training was helpful in decreasing cognitive biases in patients diagnosed with schizophrenia. In more detail, the training helped them in avoiding fast, heuristically driven decisions, such as dichotomous thinking and jumping to conclusions. Royce et al. (2019) further argued that metacognitive skills and practice could be used to increase diagnostic accuracy by having their physicians become aware of their potential cognitive biases. In the same vein, we hypothesized that the probability of selecting risky decisions, namely resulting from the HHF, would be smaller in individuals who had a higher level of metacognitive accuracy, or what we here call metacognitive awareness.

In particular, we were interested in seeing if metacognitive awareness would be of help in avoiding bias in the context where risky decisions can be critical, as in gambling. Unlike athletics, where the hot hand phenomenon can be uplifting in some contexts, as said above, gambling scenarios are those where the hot hand phenomenon can only lead to bad decisions. A few studies have shown that, in fact, metacognition has been related, negatively, with problem gambling (or pathological gambling). Brevers et al. (2014) argued that impaired metacognitive awareness of problem gamblers, as measured by inaccurate judgments of performance, was related to increased problem gambling. Similarly, Lindberg et al. (2011) also found that there was a significant negative correlation between metacognition, as measured by the Metacognition Questionnaire 30 scale (MCQ-30), and problem gambling. 

These researchers suggested that metacognitive intervention can be used to prevent problem gambling or to decrease pathological gambling symptoms. Recently, using a real-world application, Gehlenborg et al. (2021) developed a metacognitive training program for problem and pathological gambling (gambling-MCT) which included eight modules that trained participants to learn about probabilities, fallacies, and so on. (e.g., near misses and the gambler’s fallacy). Their program was successful in that it succeeded in decreasing the frequency of problem gambling. That is, the program significantly decreased gambling thought, gambling behavior, and gambling attitudes and beliefs, as compared to baseline measures. Given these prior positive results, we also predicted that metacognitive awareness would be a benefit, in that it would be negatively related to usage of the hot hand heuristic and, consequently, allow individuals to avoid the HHF.

## 3. The Current Study

In this study, we asked the following questions: First, can the HHF give rise to making riskier decisions? Second, can metacognitive awareness help in avoiding the fallacy? To address these questions, we observed the relationship between using the HHF and the number of suboptimal selections when faced with a gamble. Furthermore, we believed that thinking about one’s own thinking or one’s self-reflective process would be helpful in reducing the tendency to fall for the HHF, and thus, we predicted that metacognitive awareness would be negatively related to the frequency of succumbing to the HHF, particularly when the hot hand heuristic led to the riskier option. Finally, we observed whether the HHF would always be “irrational”. To test this, we compared predictions following two types of streaks: win streaks and loss streaks. This was because we recognized that the hot hand heuristic may not always lead to a suboptimal decision—for instance, after a series of negative events such as loss streaks, using the hot hand heuristic could lead to an optimal selection as an individual might avoid selecting this gamble.

## 4. Pilot Experiment

Our goals were straightforward. We set out to look at a very particular question: How much people tended to fall into fallacies and whether that usage would change based on metacognitive awareness. To address our question, we needed to understand people’s baseline tendencies of using heuristics, if at all. At the outset, we considered that two common heuristics that individuals might tend to use would likely lead to either the GF or the HHF. We did not have a sense of which would be more common in our case and, therefore, began with an examination for baseline tendencies. For this reason, we refer to this study as a pilot experiment. We also hoped that the current findings would be a jumping off point for further research where each fallacy on its own could be understood in various contexts. Finally, as is described in the next section, we discovered that the participants in our study were much more likely to abide by the hot hand heuristic, rather than succumbing to the GF. Thus, we decided to focus solely on the HHF in the current experiment. As a result, while we ended up analyzing fewer participants than we had tested, we were satisfied in being able to hone in on just the HHF, which had been the less-studied one of the two fallacies. 

### 4.1. Participants

Participants in the study were 54 undergraduate students taking Introductory Psychology courses at a US college. However, from preliminary analysis, we found that an insufficient number of them (*n* = 13) displayed a tendency for using the GF. Thus, we eliminated those 13 participants and chose to focus on only those participants that tended to use the hot hand heuristic. As a result, the participants in the current pilot experiment were 41 individuals between the ages of 18 to 27 (*M* = 19.49, *SD* = 1.63); 92.7% were female. All participants provided informed consent and received experimental credit for their participation. This study was approved by the College’s Internal Review Board (IRB) 2223-0530-062, and all methods were carried out in accordance with ethical guidelines.

### 4.2. Materials

Three measures were used: Iowa gambling task, Gambling Task A, and Gambling Task B. Each are described below.

Iowa Gambling Task. While the Iowa gambling task (IGT) has been frequently used to gauge decision making, it has only been used several times in an attempt to measure metacognition (Brevers et al. 2013; Persaud et al. 2007). In the original IGT (Bechara et al. 1994), individuals were presented with 4 decks of cards and told that each deck would offer a varying reward and a penalty amount. The penalty amount and the probability of the penalty were different for each deck. We created a modified Iowa gambling task to measure an individual’s metacognitive awareness level using a specific index proposed by Schraw (2009). Participants were asked to choose one of four decks labeled A, B, C, and D. If they chose either A or B, there were two possible results with different monetary values: win $100 or lose $250. If they chose C or D, there were two possible results with the same value: win or lose $50. The chances of winning were 50%. Participants completed 100 trials without any time limit. While we “divided” the 20 trials into blocks (for analysis purposes), participants were not aware of the blocks, and there were no breaks in between. Furthermore, as in the original IGT, the four decks were divided into two types: a “Good Deck” or a “Bad Deck”. Decks A and B were Bad Decks because their expected value was negative. However, Decks C and D were Good Decks because their expected value was more positive than either of the Bad Decks. After making their selection, participants were then asked to rate their confidence in their choice (on a ten-point interval scale: 10% to 100%), after which they would observe the result of their choice (i.e., the amount of money that they won or lost). As in the original IGT, the participant’s primary purpose was to acquire as much money as possible.

To measure metacognitive awareness, we calculated the Discrimination Index (DI) that had been proposed by Schraw (2009). The formula is as follows:

$$Discrimination\;Index=\frac{1}{N}\left[\sum_{i=i}^{N_{C}}\left(c_{i\;correct}\right)-\sum_{i=1}^{N_{i}}\left(c_{i\;incorrect}\right)\right]$$

In the formula, $c_{i\;correct}$ referred to the participant’s confidence rating of their choice of what we have decided to label a “correct” answer (i.e., Good Deck), while $c_{i\;incorrect}$ referred to the confidence rating regarding an “incorrect” answer (i.e., Bad Deck). This index had two directions: positive DI or negative DI. A positive DI referred to a high ability to discriminate correct from incorrect answers, indicating a higher level of metacognitive awareness; a negative DI referred to a low ability to discriminate, indicating a lower level of metacognitive awareness. These metacognitive awareness levels by DI were our moderating variable.

Gambling Task A. A new gambling task, named Gambling Task A, was created to measure each participant’s baseline for exhibiting a hot hand tendency. During the task, a gamble with a 50 percent chance of winning was offered, and participants were asked to choose one of two alternatives: bet or no bet. Before doing so, however, they also observed the prior six results of the gamble, as in the following sample pattern: W-L-W-W-W-W, where Ws and Ls represented wins and losses, respectively. Participants completed 64 trials without any time limit or breaks. We coded participants’ responses in a specific way to observe how often they followed the HHF. For example, if they chose “no bet” with prior results looking like “L-W-L-L-L-L”, responses were coded as 1 to indicate the presence of the HHF, as losses seemed to be “hot”. By contrast, if they chose to “bet,” responses were coded as 0, indicating that they did not believe that losses were hot. Afterwards, the participant’s Tendency Index (TI) was calculated by dividing the number of times the HHF was followed by the total number of trials. An index closer to 1 indicated a stronger hot hand tendency, whereas an index closer to 0.5 indicated a weaker hot hand tendency. Finally, we split participants into two tendency level groups by using the median of 0.75, resulting in the following groups: the weak group (TI < 0.75) and the strong group (TI ≥ 0.75). This was used as our independent variable, called the “Tendency Level Group”. Note that a TI of less than 0.5 indicated a tendency for using the GF, which we did not look at in our analysis.

Gambling Task B. We revised the IGT we created to also act as our dependent variable, measuring the frequency of selecting a suboptimal alternative (namely, risk selection driven by HHF). In this task, participants were asked to choose one of two cards labeled “Card A: Low risk-Low return” and “Card B: High risk-High return”. Overall, the procedure was similar to the IGT. Participants completed 100 trials without time limits. In addition, as in Gambling Task A, they also observed the prior results of Card B, which included streaks such as “W-L-L-W-W-W,” before making their choice. If they chose Card A, they would win $40 or lose $40; if they chose Card B, they would win $80 or lose $200. Card A was the more optimal choice as each option had an equal—50 percent—chance of winning. In other words, participants would lose more money if they selected Card B. We set the number of selecting Bad Card B as a dependent variable in this study. 

Finally, we included one more dependent variable, labeled “Streak Valence,” which would indicate either a “win streak” (e.g., W-W-W-L-W-W) or a “loss streak” (e.g., L-L-L-W-L-L). This was to see whether wins or losses would mean something different for the participant. For instance, if a participant observed a “win streak” occurring for a relatively riskier option, then they may be more likely to select that risky option, especially if they tended to use the hot hand heuristic already. On the other hand, if that same participant were to observe a “loss streak”, they may be more likely to avoid that risky option. Statistical tests for each dependent variable, including the streak valence, would allow us to check whether the HHF would always lead to a non-optimal decision. 

### 4.3. Procedure

All procedures were administered through a computer using E-prime 3 software. Participants were first asked to complete the IGT. Afterwards, Gambling Task A was conducted to measure each participant’s HHF tendency level. Once they completed Task A, participants completed Gambling Task B. The entire procedure took approximately 30 min. At the end, participants were debriefed and dismissed.

## 5. Results

### 5.1. Descriptive Statistics and Correlation Analysis

We first calculated the mean, median, standard deviation, skewness, and kurtosis for the variables from each task. The results of these descriptive statistics are shown in Table 1. As can be seen, the mean metacognitive awareness level was 5.888, which indicated that participants were fair when it came to distinguishing between correct and incorrect answers on the IGT. The means of each tendency level group were 0.569 (a relatively weak hot hand tendency) and 0.889 (a stronger hot hand tendency). An independent-sample *t*-test revealed that the strong group was significantly more likely to follow the hot hand heuristic as compared to the weak group ($t\left(39\right)=-12.050,\;p<.001$). The means of the risk selections were 0.506 and 0.365 for win streaks and loss streaks, respectively. Split by strong group/weak group, the means of the risk selection for the win streaks were 0.418 (weak group) and 0.589 (strong group); for the loss streaks, they were 0.383 (weak group) and 0.348 (strong group). We also considered whether the distribution of each variable followed a normal distribution by calculating skewness and kurtosis. To satisfy normality using a criterion of skewness less than the absolute value of 2 and kurtosis less than absolute value of 7 (Kim 2013; West et al. 1995), we found that all variables were considered to follow a normal distribution.

We further conducted a correlation analysis between the variables. The results are present in Table 2. As shown in the table, metacognitive awareness and tendency level were not significantly correlated (r=−.151, p>.05). However, there was a marginal negative correlation between metacognitive awareness and risk selection for the win streak condition (r=−.282, p=.07) and a borderline positive correlation between tendency index and risk selection for the win streak condition (r=.303, p=.05). In addition, metacognitive awareness was negatively correlated with risk selection for the loss streak condition (r=−.346, p<.05). However, the tendency index was not correlated with risk selection for the loss streak condition (r=−.030, p>.05), nor was the correlation between risk selections (r=.259, p>.05). 

### 5.2. Risk Selection by Metacognitive Awareness on the Iowa Gambling Task

To observe whether metacognitive awareness was appropriately measured by the IGT, we used a 2 × 5 factorial ANOVA where the first variable was metacognitive awareness as a between-subjects factor and block as a within-subject factor. The median for the metacognitive awareness was used to divide participants into two groups: “Low Meta” (M=−7.435, SD=7.885) versus “High Meta” (M=18.576, SD=16.508). Block was divided into five sections of 20 trials each, sequentially, for the IGT.

**Figure 1:**
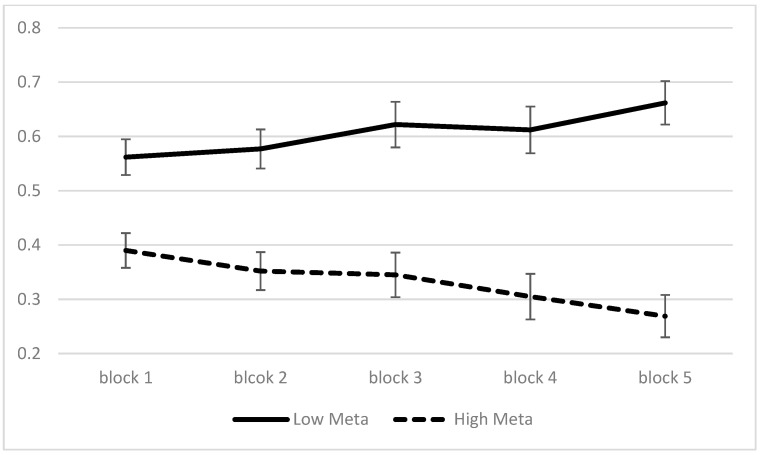
Risk Selection on the Iowa Gambling Task by Block.

Descriptive statistics of risk selection by variable is presented in Table 3. The results revealed that there was a significant interaction effect on risk selection ($F=3.902,\;p<.01,\;\eta^{2}=.09$). In addition, there was a significant main effect of metacognitive awareness ($F=49.854,\;p<.001,\;\eta^{2}=.56$). The High Meta group’s risk selection (M=.332, SE=.027) was significantly lower than that of the Low Meta group (M=.608, SE=.028). However, there was no significant main effect of block. A graph to visualize the interaction effect between independent variables is shown in Figure 1. We further conducted simple-comparison analyses to see how the effect of one independent variable depended on the level of another independent variable. Specifically, the High Meta group’s risk selection was significantly less than that of the Low Meta group, for all blocks (p<.05). In addition, the High Meta group’s risk selection in Block 5 (M=.269, SE=.039) was significantly less than that in Block 1 (M=.391, SE=.032; p<.05), and the High Meta group’s risk selection in Block 4 (M=.305, SE=.042) was also marginally less than that in Block 1 (M=.391, SE=.032; p=.06). On the flipside, the Low Meta group’s risk selection in Block 5 (M=.663, SE=.040) was marginally greater than that in Block 1 (M=.563, SE=.033; p=.05). These results suggest that utilizing the IGT to measure metacognitive awareness was suitable in this study.

### 5.3. Effects of Metacognitive Awareness and Tendency Level on Risk Selection for the Win Streak Condition

In this section, our goals were as follows: (1) to observe whether the variables significantly affected risk selection (dependent variable); (2) to observe whether there was a significant interaction effect (moderating effect) between the variables on risk selection. To accomplish these purposes, we used SPSS PROCESS Macro model 1 (4.2 version), which was proposed by Hayes (2017). We set metacognitive awareness as the independent variable, tendency level as the moderating variable, and risk selection by win streak condition as the dependent variable. The metacognitive awareness was centered around the mean. In addition, we applied the sample size as a bootstrap of 5000 and set the confidence interval at 95%. 

We begin by presenting the results for the win streak condition. As is shown in Table 4, the regression model was significant (${F=4.232,p<.05,\;R}^{2}=.255$). Metacognitive awareness had a significant negative effect on one’s risk selection for the win streak condition (B=−0.007, p<.05). Tendency level had a positive effect on risk selection (B=0.162, p<.05). The interaction effect between metacognitive awareness and tendency level was significantly positive on risk selection (B=0.009, p<.05). 

**Figure 2:**
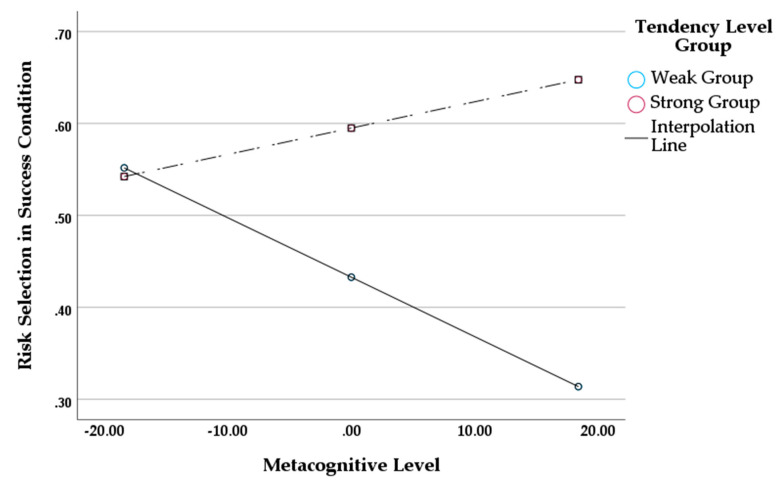
Interaction Effect between Metacognitive Awareness Level and Tendency Level on Risk Selection for the Win Streak Condition.

We further conducted a Johnson–Neyman analysis to see the significance of conditional effects of tendency level group at each point of metacognitive awareness. The results of this analysis are presented in Table 5. As is shown, the effects of tendency level were significant beyond the specific value of metacognitive awareness of −1.161. In other words, those in the strong group had positive effects on risk selection as they were more accurate in metacognitive discrimination (>−1.161). In addition, differences between tendency level groups (i.e., the effects of tendency level group) increased as metacognitive awareness increased. That is, those in the weak group were less likely to choose risky options compared to those in the strong group as metacognitive awareness increased. A graph to visualize the interaction effect is presented in Figure 2. Unlike the strong group, the weak group was less likely to select the risky option (i.e., the bad deck) as one’s metacognitive awareness increased.

### 5.4. Effects of Metacognitive Awareness and Tendency Level on Risk Selection for the Loss Streak Condition

**Figure 3:**
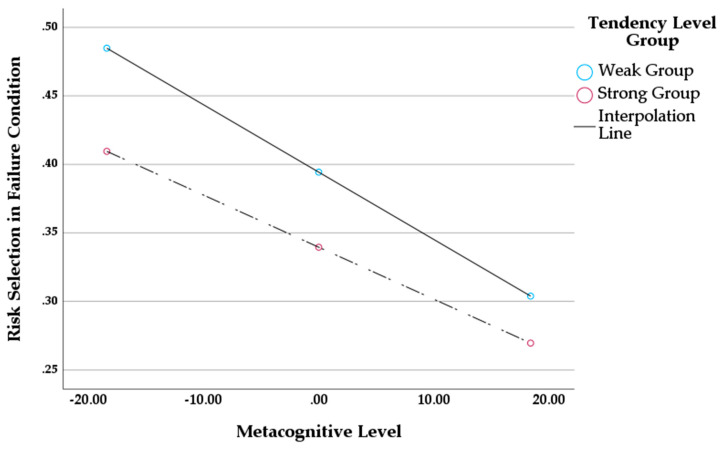
Interaction Effect between Metacognitive Awareness and Tendency Level on Risk Selection for the Loss Streak Condition.

We next moved on to the results for the loss streak condition. The goals and analysis method were same as above. As is shown in Table 6, the regression model was not significant (${F=1.936,p>.05,\;R}^{2}=.136$). Metacognitive awareness had a significant negative effect on risk selection for the loss streak condition (B=−0.005, p<.05). However, tendency level and the interaction effect between the variables were not significant (p>.05). As can be seen in Figure 3, regardless of tendency level, participants were less likely to choose the bad deck as metacognitive awareness increased.

## 6. Discussion

In this study, we investigated the relationship between metacognitive ability and likelihood of succumbing to the HHF. To accomplish this, we presented various risky options accompanied by prior “patterns”. By comparing to each participant’s baseline tendency for using the hot hand heuristic, we hypothesized that those high on the tendency marker would select the riskier option more frequently but differently in the streak valence conditions. Those with a strong baseline tendency would select the riskier option more frequently after winning streaks but would reverse their selection after a loss streak. At the same time, however, we predicted that those higher in metacognitive awareness would be less likely to use the heuristic, presumably knowing to avoid what would be a fallacy. Our results showed an interesting complexity: our predictions were supported but only partially. For winning streaks, participants high on metacognitive awareness exhibited a decreased level of risky selections, especially if they were already less prone to using the hot hand heuristic. However, for those with a strong baseline tendency to follow the heuristic, a high level of metacognitive awareness could not protect against risky selections. For loss streaks, we found that a higher level of metacognitive awareness seemed beneficial: participants from both the weak and strong tendency groups backed off the hot hand heuristic, perhaps avoiding a fallacy.

The latter finding above, where participants were able to avoid the HHF regardless of whether they tended to use the heuristic or not, might not be too surprising, given what we know of prospect theory (Kahneman and Tversky 2013). According to this theory, people perceive losses to be more punishing than gains are rewarding and, therefore, make decisions in the direction of avoiding losses. It may be that with gambles that result in loss streaks, the “punishment” (of losing) is significantly more salient than any rewards or other factors. However, what is interesting is that the perception of loss-aversion may require some amount of deliberate or conscious attention. This could explain the current data indicating that when a participant is more aware or better able to discriminate between results, their tendency to use the hot hand heuristic becomes less significant. Rather, the losses become apparent, and presumably, the use of the heuristic may have been “turned off”. 

The other piece of our data, where participants at different baseline tendency levels diverged when it came to levels of metacognitive awareness for win streaks, is complex but perhaps more interesting to think about. It was found that those who had a weaker general tendency to use the hot hand heuristic also were further less likely to use the heuristic as levels of metacognitive awareness rose. This seems fairly logical: the not-so-determined-hot-hand-users “became” more cautious about succumbing to this fallacy when they were more metacognitively accurate. But what about those who were relatively more faithful to the heuristic at baseline? Even with a higher level of metacognitive awareness, their use of the hot hand heuristic did not falter. On the contrary, numerically, it seemed to become stronger. At first glance, it seems that metacognitive awareness may not always be effective at helping individuals avoid fallacies. 

On the other hand, perhaps in the latter instance, the hot hand heuristic might be considered to be a non-fallacy. We have seen that the hot hand is, in general, something that we long for. While it may certainly lead to a fallacy that could be detrimental in gambling, the same is not true in all situations. Turn on any sporting event, and it is easy to find someone—sportscasters, fans, and even those sitting on the side of the opponent team—shout out the player with the “hot hand”. It is likely that those who have a strong tendency to follow the hot hand heuristic are likely to be ones who truly believe that it is motivating, whether it leads to a fallacy or not. Given that these data were collected on college-aged students, one can imagine that individuals in this category developed their hot hand inclinations while cheering for their school team and not gambling at the casinos. Thus, those who are loyal to the hot hand and aware of the winning streaks are likely to continue to hold on to what is hot.

At the same time, we recognize that our data describe a very particular group of participants—young adults that consist largely of one gender. The data may look vastly different in various participant pools; not only might one see stronger hot hand tendencies, but on the flipside, we might be forced to analyze data for those who tended to succumb to the GF. What we do know is that the level of tendency of using a particular heuristic seems to lead to different interactions when it comes to metacognitive awareness. Thus, research in different contexts is crucial for a fuller understanding of the fallacies and our goals to decrease them appropriately. 

Finally, there may be other variables that could explain the correlation between metacognitive awareness and risk selections. We found that metacognitive awareness did not lead to a unidirectional change in behavior. We had thought that, on the whole, a higher level of awareness would be associated with a smaller dependence on heuristics, especially given that those with high metacognitive awareness have been thought to be less impulsive (Miller et al. 2021), and greater levels of self-regulation (Arslan 2014). We found that for those with a strong tendency to use the heuristic held strong—and numerically increased—in the use of the heuristic. Thus, it would be beneficial to re-investigate the relationship with other variables, focusing, perhaps, on motivational aspects of the hot hand. 

The current study aimed to understand the relationship between the HHF and risky decision-making and moreover to see whether metacognitive awareness would be helpful in avoiding the hot hand heuristic. The data indicated a level of complexity, in that participant responses will depend on both their baseline tendency to use the heuristic as well as the prior streak valence. While our hypotheses were partially supported—for the most part, metacognitive awareness was associated with a lower level of the HHF—we were intrigued to find that those with a strong tendency to use the heuristic continued to hold on to the heuristic, even when metacognitive awareness was at a higher level. Thus, there may be other factors that seem to encourage belief in the hot hand, a notion that might warrant new research and, perhaps, the term “fallacy” to be reconsidered. 

## Figures and Tables

**Table 1:** Descriptive Statistics of Metacognitive Awareness, Tendency Level, and Risk Selections.

Variables		N	Mean	Median	SD	Skewness	Kurtosis
Metacognitive Awareness		41	5.888	1.900	18.414	.906	.615
Tendency Level Groups (Tendency Index)	Weak Group	20	0.569	0.545	0.077	.889	−.514
	Strong Group	21	0.889	0.932	0.092	−.389	−1.498
	Total	41	0.733	0.750	0.182	.074	−1.522
Risk Selection (Win Streak)	Weak Group	20	0.418	0.450	0.297	−.143	−1.432
	Strong Group	21	0.589	0.600	0.203	−.321	.361
	Total	41	0.506	0.567	0.265	−.493	−.606
Risk Selection (Loss Streak)	Weak Group	20	0.383	0.417	0.237	−.147	−.587
	Strong Group	21	0.348	0.367	0.234	.595	1.000
	Total	41	0.365	0.367	0.233	.220	−.083

The descriptive data for each task: The value of metacognitive awareness is taken from the IGT; the values of tendency level groups are taken from Gambling Task A; the values of risk selections are taken from Gambling Task B.

**Table 2:** Correlations between Metacognitive Awareness, Tendency Level, and Risk Selections.

	1	2	3	4
Metacognitive Awareness	1	(.347)	(.074)	(.027)
Tendency Index	−.151	1	(.054)	(.852)
Risk Selection (Win streak)	−.282	.303	1	(.101)
Risk Selection (Loss streak)	−.346 *	-.030	.259	1

Values in parentheses indicate the *p*-values of correlational analysis between the variables. * indicates p<.05.

**Table 3:** Descriptive Statistics of Risk Selection between Metacognitive Awareness and Block on the Iowa Gambling Task.

	Block 1	Block 2	Block 3	Block 4	Block 5	Total
Low Meta N=20	0.563 (0.119)	0.578 (0.133)	0.623 (0.144)	0.613 (0.149)	0.663 (0.123)	0.608 (0.089)
High Meta N=21	0.391 (0.171)	0.352 (0.181)	0.345 (0.222)	0.305 (0.224)	0.269 (0.222)	0.332 (0.151)
Total N=41	0.474 (0.170)	0.462 (0.195)	0.481 (0.233)	0.455 (0.244)	0.461 (0.268)	0.466 (0.186)

Values indicate means and standard deviations for risk selection.

**Table 4:** PROCESS Macro Model 1 Results for the Win Streak Condition.

Model	DV = Risk Selection (Win Streak)					
	Coefficient (*B*)	SE	*t*	*F* *(df 1, df 2)*	$R^{2}$	$∆R^{2}$
Constant	.433	.053	8.110 *	4.232 * (3, 37)	.255	.089
Tendency Level Group	.162	.075	2.169 *			
Metacognitive Awareness	−.007	.003	−2.607 *			
ML × TLG	.009	.004	2.105 *			

* indicates p<.05.

**Table 5:** Conditional Effects of Tendency Level Group by Metacognitive Awareness.

Metacognitive Awareness	Effect	SE	*t*	LLCI	ULCI
−33.688	−0.152	0.164	−0.926	−0.484	0.180
−29.428	−0.112	0.147	−0.761	−0.410	0.186
−25.168	−0.072	0.131	−0.550	−0.338	0.194
−20.908	−0.033	0.116	−0.280	−0.268	0.203
−16.648	0.007	0.103	0.069	−0.201	0.215
−12.388	0.047	0.091	0.516	−0.137	0.231
−8.128	0.087	0.082	1.061	−0.079	0.252
−3.868	0.126	0.076	1.661	−0.028	0.280
−1.161	0.151	0.075	2.026	0.000	0.303
0.392	0.166	0.075	2.215 *	0.014	0.318
4.652	0.206	0.078	2.620 *	0.047	0.365
8.912	0.245	0.086	2.849 **	0.071	0.420
13.172	0.285	0.097	2.942 **	0.089	0.481
17.432	0.325	0.110	2.956 **	0.102	0.547
21.692	0.364	0.124	2.930 **	0.112	0.616
25.952	0.404	0.140	2.889 **	0.121	0.687
30.212	0.444	0.156	2.842 **	0.127	0.760
34.472	0.483	0.173	2.796 **	0.133	0.834
38.732	0.523	0.190	2.752 **	0.138	0.908
42.992	0.563	0.208	2.711 *	0.142	0.983
47.252	0.603	0.225	2.675 *	0.146	1.059
51.512	0.642	0.243	2.641 *	0.150	1.135

* indicates p<.05 ** indicates p<.01.

**Table 6:** PROCESS Macro Model 1 Results for the Loss Streak Condition.

Model	DV = Risk Selection (Loss Streak)					
	Coefficient	SE	*t*	*F* *(df 1, df 2)*	$R^{2}$	$∆R^{2}$
Constant	.394	.051	7.780 *	1.936 (3, 37)	.136	.002
Tendency Level Group	−.055	.071	−0.771			
Metacognitive Awareness	−.005	.002	−2.088 *			
ML × TLG	.001	.004	.264			

* indicates p<.05.

## Data Availability

The data for this experiment can be found at the following site: https://osf.io/hkmex/?view_only=88a244ecbbd745ed942794717e407189 (accessed on 9 July 2023).
